# Supplementary material for: Transcriptome Characterization and Gene Changes Induced by Fusarium solani in Sweetpotato Roots
Source: Genes (Basel). 2023 Apr 25;14(5):969. doi: 10.3390/genes14050969 (PMC10218436; doi:10.3390/genes14050969)
Supplement: Supplementary file 1 [file genes-14-00969-s001.zip › Supplementary data to this article.pdf]

Supplementary data to this article:

Additional file 1:

Figure S1. Sample correlation heat map.

Additional file 2:

Table S1. Data filtering statistics

Additional file 3:

Figure 2S. Bubble plot of the KEGG pathway enrichment of DEGs. The top 20 with the lowest Q value of enriched pathways for comparisons: A: CK-32 vs. T-6h, B: CK-32 vs. T-24h, C: CK-32 vs. T-3d, D: CK-32 vs. T-5d. Bubble color and size correspond to the Q value and gene number enriched in the pathway. The rich factor indicates the ratio of the number of DEGs mapped to a certain pathway to the total number of genes mapped to this pathway.

Additional file4:

Table S2 Related genes and pathways in plant-pathogen interaction

Table S1. Data filtering statistics

Samples	RawData (bp)	CleanData(%)	Q30	Total	TotalMapped(%) /Sweetpotato	TotalMapped(%) /*Fusarium solani*
CK-32-1	64427836	64368904 (99.91%)	9073817079 (93.89%)	63216130	48523437 (76.76%)	
CK-32-2	67448358	67381806 (99.90%)	9574163424 (94.63%)	64493084	49919241 (77.40%)	
CK-32-3	75132248	75007404 (99.83%)	10455804188 (92.78%)	73695904	56561942 (76.75%)	
T-6h-1	72722906	72613742 (99.85%)	10238442215 (93.86%)	65060974	8043131 (12.36%)	57.21%
T-6h-2	64040800	63977486 (99.90%)	9054305795 (94.26%)	40119272	4785461 (11.93%)	56.57%
T-6h-3	73692006	73594512 (99.87%)	10056287813 (90.98%)	60484944	7615932 (12.59%)	57.42%
T-24h-1	82486622	82358066 (99.84%)	11573702850 (93.54%)	72035904	7532664 (10.46%)	0.58%
T-24h-2	75839274	75754808 (99.89%)	10707651954 (94.13%)	66444752	8941094 (13.46%)	0.58%
T-24h-3	70264758	70146802 (99.83%)	9860246945 (93.55%)	62022092	6785886 (10.94%)	4.58%
T-3d-1	75006824	74896992 (99.85%)	10475824775 (93.11%)	74243204	57016513 (76.80%)	0.59%
T-3d-2	71971622	71857110 (99.84%)	10000613841 (92.63%)	70796204	54208631 (76.57%)	1.68%
T-3d-3	68745452	68459012 (99.58%)	9636337809 (93.45%)	67674158	48180955 (71.20%)	0.45%
T-5d-1	76647188	76552916 (99.88%)	10687484001 (92.96%)	75670110	58190196 (76.90%)	57.21%
T-5d-2	78803984	78723616 (99.90%)	11123411184 (94.10%)	77046624	58378032 (75.77%)	56.57%
T-5d-3	66434978	66334630 (99.85%)	9217749586 (92.50%)	65710952	49970730 (76.05%)	57.42%

Table S2. Related genes and pathways in plant-pathogen interaction

pathway/ describe (K_id)	comparisons	Differentially expressed genes/down	Differentially expressed genes/up
Fungal PAMP/CPK; calcium-dependent protein kinase (K13412)	CK-32 vs. T-6h	G1629, G25763, G27922, G27924, G29161, G3795, G4098, G4116, G44256, G47022	G23385, G23394, G26201, G26202, G26207, G33768, G42600, G44834
	CK-32 vs. T-24h	G1629, G4112, G3795, G4098, G3406, G4116,G47022, G25763	G26207,G44834, G33768, G23385, G42600, G26202
	CK-32 vs. T-3d	G3795, G1629, G27922, G27924, G48602, G40200, G4112, G7779, G4116, G27539, G4098, G3271, G3406, G25763, G47022,	G26292, G44834, G26201, G26202, G26207, G23385, G23394, G33768, G42600
	CK-32 vs. T-5d	G3795, G27922, G27924, G1629, G40200,G4112, G4098, G4116, G25763,	G26207, G33768, G42600, G23385
Fungal PAMP/Rboh; respiratory burst oxidase (K13447)	CK-32 vs. T-6h	G41782, G46408,G46406, G22301,G22322,	G225, G3921, G3924, G4567, G47639, G47642, MSTRG.314
	CK-32 vs. T-24h	G46408,G22301,G41782, G46406, G22322,	G47642,G3924, G4567, G225, MSTRG.314, G3921, G47639
	CK-32 vs. T-3d	G41782, G22301,G46406, G22322,G46408,G39844	G3924,G47642,G4567, G225, G47639
	CK-32 vs. T-5d	G41782, G22301,G22322,G46408,G46406	G225, G3921, G3924, G4567, G47642, MSTRG.314
Ca^{2+}/CNGC; cyclic nucleotide gated channel, plant (K05391)	CK-32 vs. T-6h	G28419, G28433, G5820, G36125, G23262, G36119, G42426	G13410, G10205, G7849,G7852
	CK-32 vs. T-24h	G28419, G23262	G13410, G10205, G7852
	CK-32 vs. T-3d	G28419, G23251, G23262, G5820, G42426	G7512, G7849,G7852

	CK-32 vs. T-5d	G5820, G5818	G28649
Ca^{2+}/CALM; calmodulin (K02183)	CK-32 vs. T-6h	G36840, G34063, G33741	G34963, G34971, G35048, G35077
	CK-32 vs. T-24h	G34063, G33741	G34963, G34971, G35077, G35048, G5856
	CK-32 vs. T-3d	G36840, G34063, G33741, G35044	G35077, G34971, G35048
	CK-32 vs. T-5d	G36840, G34063, G33741	G34971, G35048, G35077,G16427
Ca^{2+}/calcium-binding protein CML (K13448)	CK-32 vs. T-6h	G5032, G42522, G5074, G19413, G8847, G24929, G26358, G26360, G22517	G25638, G25967, G25986, G6338
	CK-32 vs. T-24h	G20662, G19413,G5074,G24929,G26358,	G25967,G25986, G6338
	CK-32 vs. T-3d	G5074, G19413, G5032, G24929, G42522, G8847, G25638, G25639, G26358, G1536, G3444, G6128	-
	CK-32 vs. T-5d	G5032, G20662, G5074, G19413, G8847,G24929, G26358, G3444, G25639, G42522	-
Ca^{2+}/NOA1; nitric-oxide synthase, plant (K13427)	CK-32 vs. T-6h	G6611, G6636, G6639	-
	CK-32 vs. T-24h	G6611, G6639	-
	CK-32 vs. T-3d	G6611, G6636, G6639, G6695	-
	CK-32 vs. T-5d	G6611, G6636, G6639	-
