# Supplementary figures and images for: Transcriptome Characterization and Gene Changes Induced by Fusarium solani in Sweetpotato Roots
Source: Genes (Basel). 2023 Apr 25;14(5):969. doi: 10.3390/genes14050969 (PMC10218436; doi:10.3390/genes14050969)

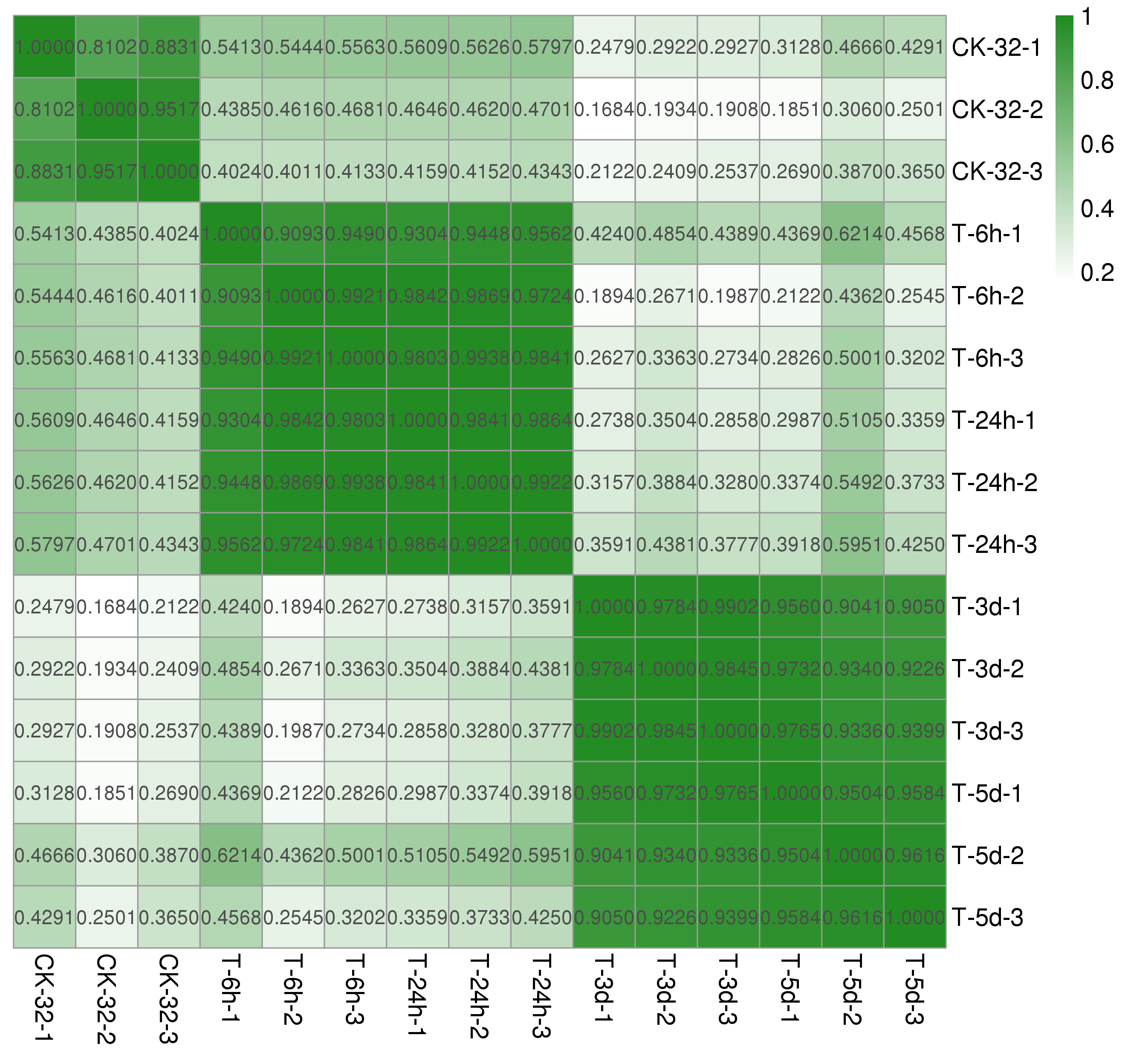

Supplement: Supplementary file 1 [file genes-14-00969-s001.zip › Figure S1.png]

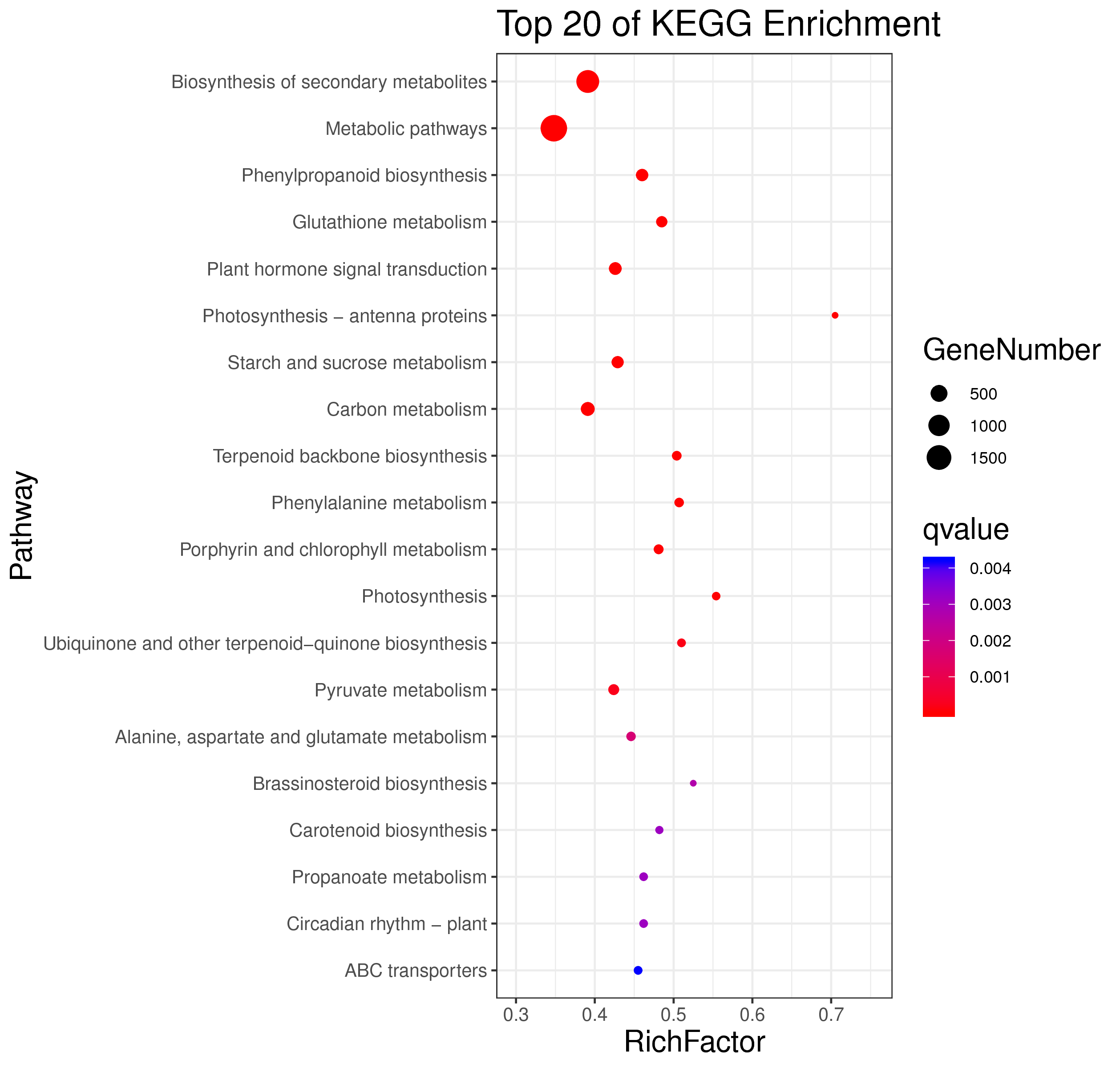

Supplement: Supplementary file 1 [file genes-14-00969-s001.zip › Figure S2 A.png]

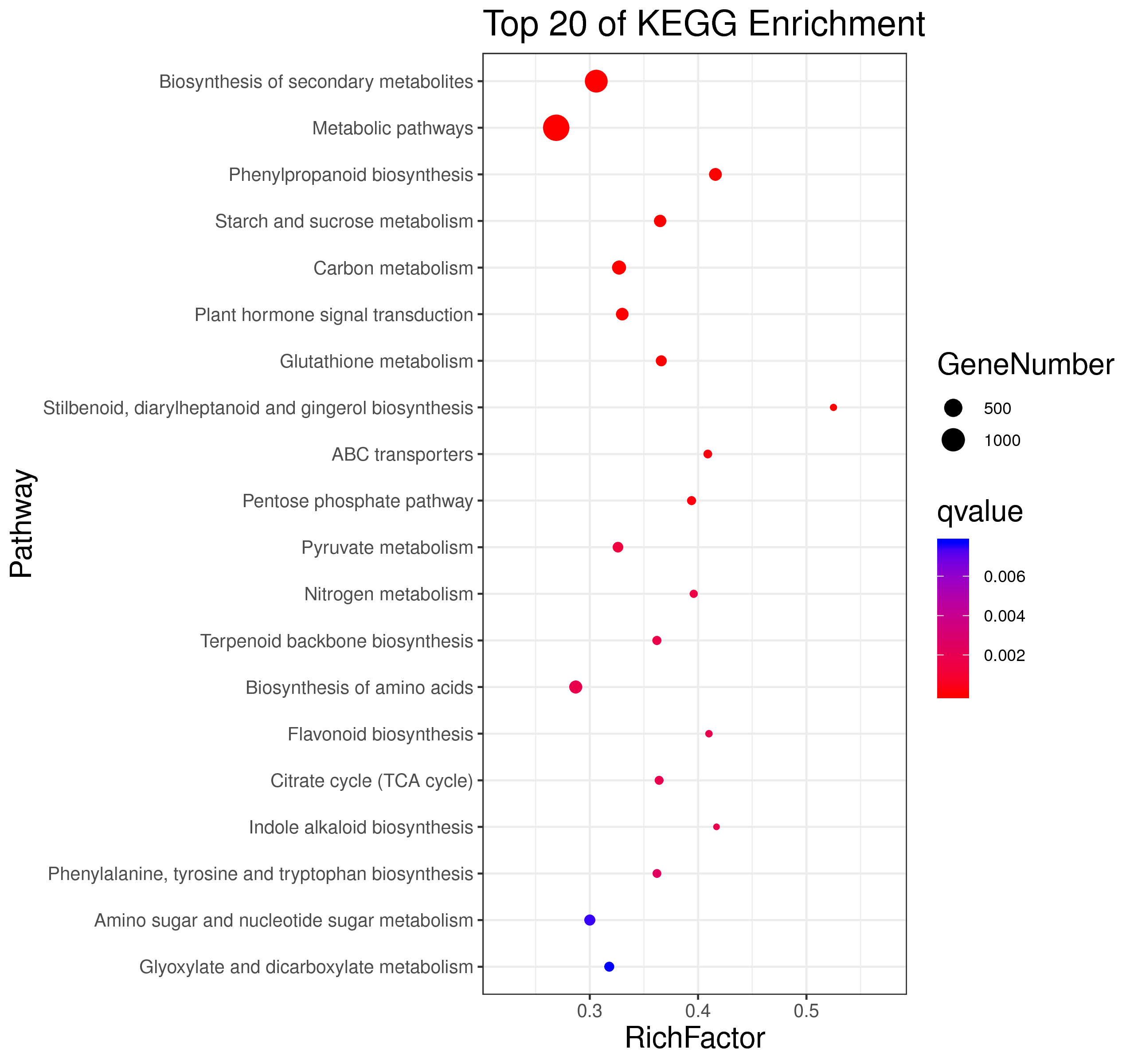

Supplement: Supplementary file 1 [file genes-14-00969-s001.zip › Figure S2 B.png]

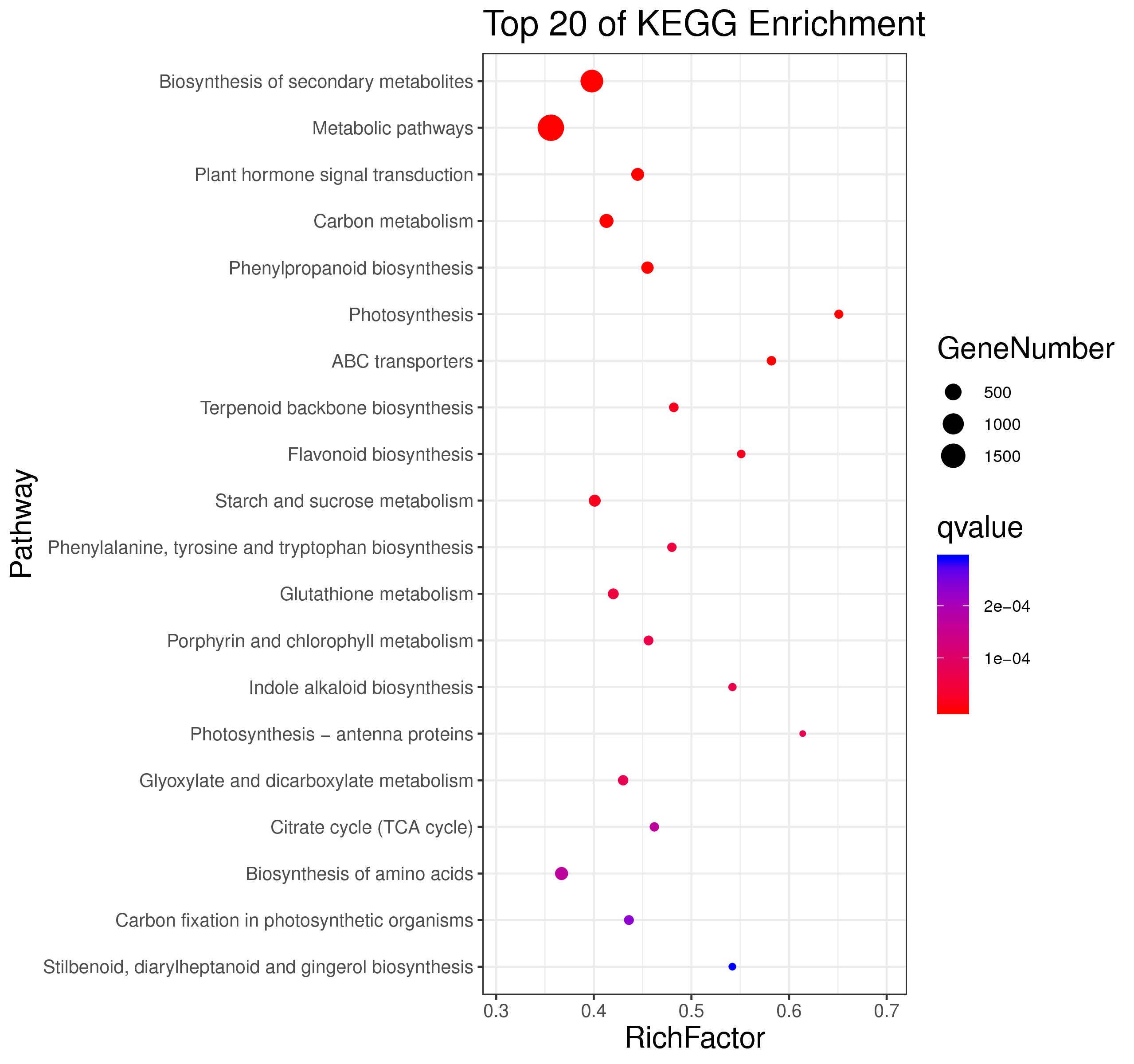

Supplement: Supplementary file 1 [file genes-14-00969-s001.zip › Figure S2 C.png]

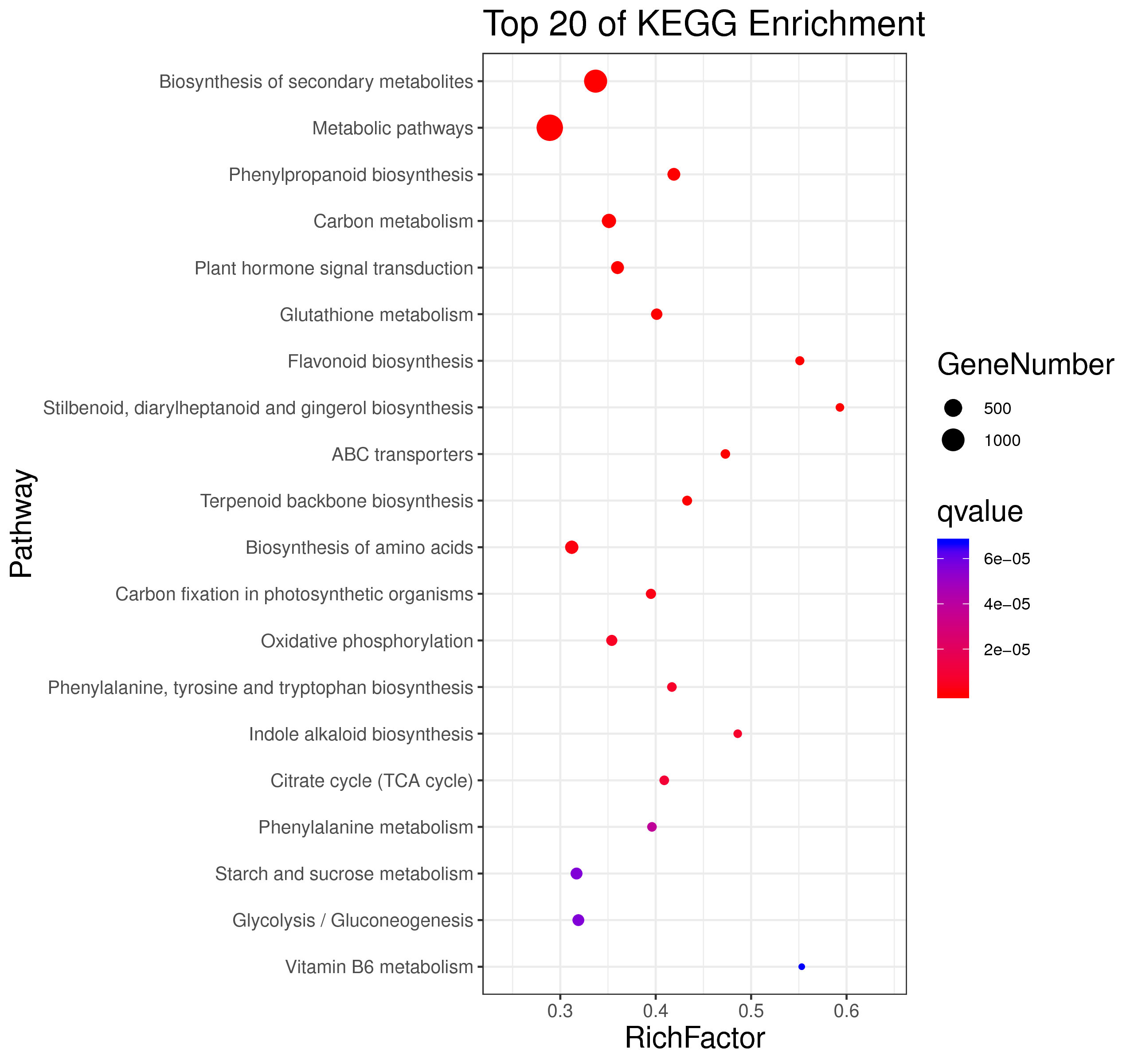

Supplement: Supplementary file 1 [file genes-14-00969-s001.zip › Figure S2 D.png]
